# EAG response and behavioral orientation of *Dastarcus helophoroides* (Fairmaire) (Coleoptera: Bothrideridae) to synthetic host-associated volatiles

**DOI:** 10.1371/journal.pone.0190067

**Published:** 2017-12-21

**Authors:** Li li Ren, Karthi Balakrishnan, You qing Luo, Stefan Schütz

**Affiliations:** 1 Beijing Key Laboratory for Forest Pest Control, Beijing Forestry University, Beijing, P. R. China; 2 Department of Forest Zoology and Forest Conservation, Georg-August-Universitat Gottingen, Göttingen, Germany; USDA Agricultural Research Service, UNITED STATES

## Abstract

*Dastarcus helophoroides* Fairmaire (Coleoptera: Bothrideridae) is an effective predatory beetle of larvae and pupae of several cerambycid beetles including *Monochamus alternatus* and *Anoplophora glabripennis*. Electroantennography (EAG) and a dynamic two-choice olfactometer were respectively used to measure the antennal and behavioral responses of both sexes to selected volatile compounds. Female and male *D*. *helophoroides* exhibited similar EAG and behavioral responses. Significant dose-dependent EAG responses in both sexes were elicited by nonanal, octanal, cis-3-hexenol, 3-carene, (R)-(+)-α-pinene, (S)-(-)-α-pinene, (R)-(+)-limonene and (S)-(-)-limonene. Female and male beetles were repelled at high concentration by cis-3-hexenol and (S)-(-)-limonene, respectively. Both sexes of *D*. *helophoroides* were significantly attracted to nonanal, cis-3-hexenol, 3-carene and (R)-(+)-limonene even at low concentrations. These compounds might be used either individually or in mixtures for developing biological control methods to attract this predatory beetle into forest stands threatened by cerambycid beetles.

## Introduction

*Dastarcus helophoroides* Fairmaire, 1881 (Coleoptera: Bothrideridae) (syn. *Pathodermus helophoroides* Fairmaire, 1881; *Dastarcus longulus* Sharp, 1885) is a predator of the larvae and pupae of important cerambycid forest tree pests such as *Monochamus alternatus* Hope 1843, *Anoplophora glabripennis* Motschulsky 1853, *Massicus raddei* Blessig & Solsky 1872, *Apriona germari* Hope 1831, *A*. *swainsoni* (Hope,1840), and *Batocera horsfieldi* (Hope, 1839), **[1–3]**. Some of these long-horned beetles are causing significant economic losses in China and other countries. For example, *A*. *glabripennis*, native to China and other Asian countries, has been introduced into North America and some European countries, devastating the hardwood forests by vigorously feeding on tree trunks. Additionally, *M*. *alternatus* is a vector of pine wilt disease by carrying its causative agent, the pine wood nematode *Bursaphelenchus xylophilus*. Therefore, *D*. *helophoroides* could be an option as a potential biological agent for controlling various cerambycid pest species.

Female *D*. *helophoroides* deposit egg clusters close to entrance holes, gallery walls and the frass of longicorn beetles larvae **[3, 4]**. The hatched predatory larvae are active and search for prey, which, once located, are paralyzed before feeding. Predators of plant feeders commonly use semiochemicals to locate their prey at both long and short distances. These semiochemicals might emanate from their prey or from trees attacked by their prey **[5–8]**. Thus, semiochemicals including monoterpenes, sesquiterpenes, and homoterpenes **[6]** can serve as indirect plant defense compounds, attracting specific predators to kill the attacking pest insects. For example, both sexes of *D*. *helophoroides* were attracted to volatiles emanating from larval tunnels and the frass of two cerambycid beetle species **[9]**. To date, only one strong attractant (R)-(+)-limonene has been identified for *D*. *helophoroides* **[10]**. However, nothing is known about the antennal detection of *D*. *helophoroides* to any previously tested compounds including (R)-(+)-limonene. Additionally, no study has examined the dose-dependent behavioral responses of *D*. *helophoroides* to any prey related volatile compounds **[10, 9]**.

The antennal sensilla of *D*. *helophoroides* have been morphologically studied and the typical chemosensitive structures (wall-pores) were found on the different types of sensilla **[11]**. Moreover, recent transcriptome analyses identified multiple chemosensory gene families in *D*. *helophoroides* that may be involved in olfaction **[12, 13]**.

The purpose of our study was to explore the antennal and behavioral responses of *D*. *helophoroides* to prey-related volatile compounds (Table 1). A total of nine volatile compounds were chosen for antennal detection and behavior experiments. Among them, 3-carene, (R)-(+)-α-pinene, (S)-(-)-α-pinene, (R)-(+)-limonene, (S)-(-)-limonene, and trans-β-caryophyllene were selected based on volatiles perceived by *Monochamus* spp. (a prey of *D*. *helophoroides*) from *Pinus* spp. trees **[5, 14]**. Additionally, octanal, nonanal, and cis-3-hexenol were selected from among volatiles released by *A*. *glabripennis* attacked *Populus nigra* **[15, 16]**. Moreover, the monoterpenes 3-carene, (R)-(+)-α-pinene, (S)-(-)-α-pinene, (R)-(+)-limonene, and (S)-(-)-limonene were reported as actively perceived by *D*. *helophoroides* from larval frass of *A*. *glabripennis*, *A*. *swainsoni* and *M*. *raddei* **[9, 10]**. Being associated with different prey species, these selected volatile compounds are potentially biologically important to *D*. *helophoroides*. We used electroantennography (EAG) **[17]** to record antennal detection and a dynamic two-choice ten track olfactometer to analyze the behavioral responses of *D*. *helophoroides* to these compounds **[18]**.

**Table 1:** Selected synthetic volatile compounds used as olfactory stimuli in the EAG and the behavioral experiments tested with predatory beetle *D*. *helophoroides*.

Chemicals	Purity %	Source	Vapor pressure Vp 25°C (mbar)	Concentrations used in the experiments	
				EAG (in paraffin mg/mg)	Behavior (in silicone oil mg/mg)
octanal	98	Tci	3.27	10^−1^–10^−6^	10^−1^, 10^−3^, 10^−5^
nonanal	95	Aldrich	0.71	10^−1^–10^−6^	10^−1^, 10^−3^, 10^−5^
cis-3-hexenol	98	Aldrich	1.39	10^−1^–10^−6^	10^−1^, 10^−3^, 10^−5^
3-carene	90	Aldrich	2.48	10^−1^–10^−6^	10^−1^, 10^−3^, 10^−5^
(R)-(+)-limonene	99	Tci	2.05	10^−1^–10^−6^	10^−1^, 10^−3^, 10^−5^
(S)-(-)-limonene	95	Tci	2.05	10^−1^–10^−6^	10^−1^, 10^−3^
(R)-(+)-α-pinene	95	Tci	4.65	10^−1^–10^−6^	10^−1^, 10^−3^
(S)-(-)-α-pinene	98	Fluka	4.65	10^−1^–10^−6^	10^−1^, 10^−3^
trans-β-caryophyllene	98.5	Aldrich	0.017	10^−1^–10^−6^	10^−1^, 10^−3^

TCI -Shanghai Development Co. Ltd., China; Aldrich- Steinheim, Germany; Fluka- Buchs, Switzerland

## Materials and methods

### Insects

Cocooned *D*. *helophoroides* pupae were obtained from a laboratory colony reared at the Natural Enemy Research Institute of the Beijing Agriculture College, China. This colony was established with *M*. *alternatus* larvae and pupae that were collected from the trunks of *Pinus massoniana* in Guangdong Province, China. No specific permissions were required for collecting *M*. *alternates* at this site because it's a common herbivorous insect in the afforestation pine plantations. The field studies did not involve endangered or protected species. In a controlled atmosphere, the *D*. *helophoroides* pupae were reared in ventilated plastic boxes at 25°C, 45% RH, and a photoperiod of light 16 h: dark 8 h. After hatching, the adults were collected, transferred to cages and fed an artificial diet **[19]**. The beetles were then imported into Germany and were maintained in a climate chamber with identical environmental conditions as in China. The age of the beetles used in the EAG and the behavioral experiments was approximately 40 days after hatching. After each experiment, actively responding beetles were sexed reliably by dissecting the reproductive organs **[20]**. Ten antennae from different adult beetles and 70–100 adult beetles of each sex were used in EAG and behavioral experiments respectively.

### Chemical stimuli

The selected compounds octanal, nonanal, cis-3-hexenol, 3-carene, (R)-(+)-limonene, (S)-(-)-limonene, (R)-(+)-α-pinene, (S)-(-)-α-pinene and trans-β-caryophyllene were diluted in paraffin oil (mg/mg) (Uvasol®, Merck, Darmstadt, Germany) and silicone oil M 200 (mg/mg) (Carl Roth GmbH + Co. KG, Germany) for EAG and behavioral experiments respectively. All compounds were obtained from commercial suppliers (Table 1).

### Electroantennogram and odor delivery

The procedure used to prepare the beetles for the electroantennographic recording is described in **[21]** and was adapted to the special requirements of *D*. *helophoroides*. Beetles were starved for 24 h before the experiments. A sharpened tungsten wire prepared by electrolytic etching was used to make a small hole in the thorax region. Later, this hole was used for the insertion of a reference glass electrode filled with Ringer’s solution in contact with an Ag/AgCl wire. The beetle was placed under a high magnification compound microscope (Leica MZ16, Leica Microsystems GmbH, Germany). To stabilize the antenna, a sharpened tungsten wire was used to hold the antennal segment in place. Then, the last antennal segment was punctured with a sharpened tungsten wire using a micromanipulator. This hole was used to insert a recording glass capillary (GB150F-8P, 0.86 × 1.50 × 80 mm with the filament; Science products GmbH, Hofheim-Deutschland) containing Ringer’s solution in contact with an Ag/AgCl wire. The EAG responses were detected through a combi-probe (INR-II; Syntech, the Netherlands). The DC potential was recorded (Universal AC/DC probe), processed and analyzed using EAG 2000 software (Syntech, Hilversum, Netherlands).

An air stimulus controller (CS55; Syntech, Hilversum, the Netherlands) was used to deliver purified air and odor. A constant flow (18 l/h) of filtered air was passed over the prepared antenna through the open end of the metal tube positioned close to the antenna. The pulse duration time was 1 s. Each volatile concentration was tested in a 1 min interval. The time interval between each volatile compound was 2 min. Ascending concentrations of each compound (10^−6^ to 10^−1^ in paraffin mg/mg) were applied to avoid olfactory adaptation. A standard green leaf volatile cis-3-hexenol at 10^−3^ (in paraffin oil mg/mg) stimulation was done at the beginning and at the end of each recording to correct for the loss of sensitivity of the antennal preparation. Similarly, a control paraffin oil stimulation was done at the beginning and at the end of each recording to subtract the blank value from the antennal responses. For each compound, EAG responses of ten antennae from different adult beetles of each sex were recorded.

### Olfactometer and behavioral test

**Fig 1:**
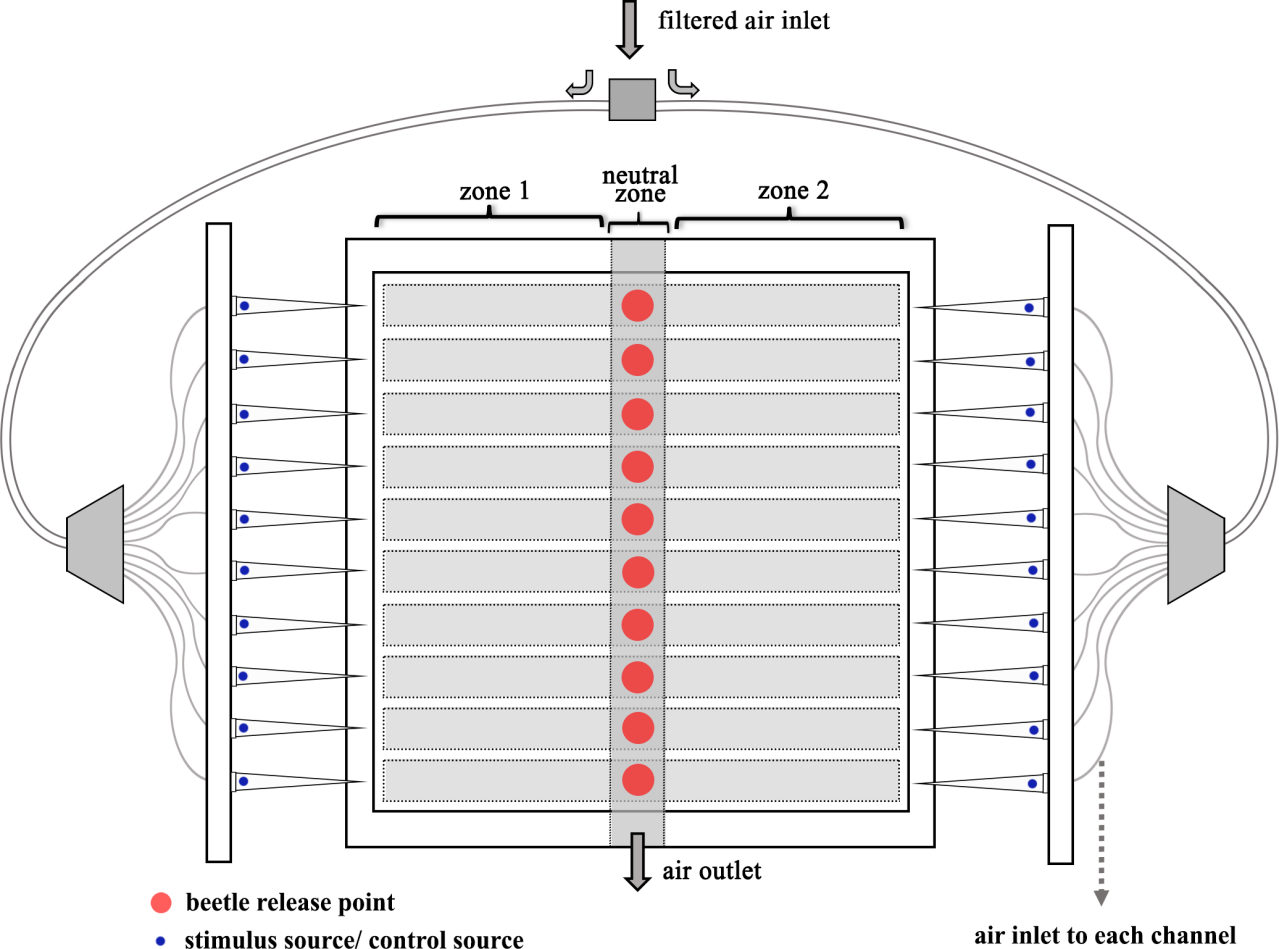
Schematic representation of the custom-built dynamic two-choice olfactometer with 10 tracks and showing the walking arena defined as zone 1, 2 and the beetle releasing point as neutral zone.

A custom-built dynamic two-choice olfactometer with 10 parallel tracks was used in the behavior test as described in **[18]**. A 40 × 180 × 170 mm olfactometer was constructed from PTFE (Polytetrafluoroethylene). The 10 parallel walking chambers were covered with a glass lid. The non-transparent PTFE-walls between tracks isolated the tested beetles from each other as well as both chemical and visual cues. Each walking chamber was 20 mm deep and 150 mm long (Fig 1). The width of each chamber was enlarged to 10 mm to allow *D*. *helophoroides* to turn around freely inside the chamber. A white LED plate (200 × 200 mm) was used as a light source beneath the walking tracks. At both ends of each walking chamber, 200 μl filter pipette tips (Sarstedt AG&CO, Nümbrecht, Germany) were inserted: 10 filter tips on one end were drenched with 65 μl of volatile compound, and 10 filter tips on the other end were drenched with pure silicone oil control. Silicone oil was used as a solvent for the behavioral assays, because of the unfavorable viscosity of paraffin oil (used in EAG) tended to obstruct the filter tips of the olfactometer. Filtered and humidified air was fed into both ends of each track at the identical constant flow rate of 0.080 L/min.

A live video tracking system, the Etho vision XT 8.0 (Noldus Information Technology, the Netherlands), was used to recognize and record the behavior of the beetles. Three compartment zones of olfactometer were defined as zone 1, zone 2, and the beetle releasing point as “neutral” zone (Fig 1). Ten insects were introduced into the olfactometer at the neutral zone in each trial, with the released beetles unable to contact each other because of the physical separation in the walking arenas. The volatile compound and the silicone oil were placed randomly in the direction of zone 1 or zone 2 in each trial. Each trial began with a 30 s accommodation phase for the beetles and lasted for 15 min. The record was valid as soon as the middle point of a beetle entered zone 1 or zone 2. The olfactometer was cleaned with methanol, and the position of the volatile compound was changed after each trial. To avoid possible influence of asymmetries in illumination on recording data, the olfactometer was turned by 180° after each true trials.

In behavioral tests, initially, we used all compounds at 10^−1^ and 10^−3^ doses. Later, those compounds showing significant differences between control and treatment zones at a dose 10^−3^ were further tested at a dose of 10^−5^ (Table 1). A control experiment with silicone oil vs. silicone oil was conducted to test the behavior of *D*. *helophoroides* without stimuli. Fifty replications were conducted for all compounds at selected concentration (10^−1^, 10^−3^, 10^−5^; Table 1).

To measure the behavioral responses of *D*. *helophoroides* beetles responding to both treatment and control zones, we calculated the total distance movement (TDM) of beetles in each trial. Beetles were released in the neutral zone of each track, and the total moving distance for 15 min in any direction in the track (to treatment zone, to control zone, returning to the neutral zone or moving to either of control and treatment zones etc.,) was calculated. Our estimation of TDM assumes that beetles respond behaviorally towards either of the tested zones (control or treatment) in each track, but it does not reveal the kinesis activity of beetles. To evaluate the attraction of beetles to tested stimuli, we calculated the beetle entering frequency (BEF) responses to treatment or control zones within 15 min. Beetles were released in the neutral zone of each track, and scored a single BEF count as the beetle moved into the treatment or control zone (may or may not reach the track end) and return to the neutral zone. As a measure of how beetles show high attraction to chemical stimuli, we calculated the beetle staying duration (BSD) responses at 5, 10, and 15 min intervals in both treatment and control zones. We scored the BSD as beetles that entered the treatment or control zone, and noted how long each beetle stayed in a particular zone across different time durations (5, 10, 15 min). Observations were stopped once the beetle exited of the zone to the releasing point (neutral zone) and resumed when the beetle entered the same zone within the assigned time duration.

### Statistical analyses

To correct the EAG responses in comparison to the paraffin oil control, the mean of the blank responses before and after the measurements were subtracted from that to elicited by the volatile compound. All EAG responses data were then normalized to cis-3-hexenol (10^−3^) as follows:

$$\frac{A-\frac{EAG(ctl1)+EAG(ctl2)}{2}}{\frac{EAG(std1)+EAG(std2)}{2}-\frac{EAG(ctl1)+EAG(ctl2)}{2}}$$

where A is the amplitude (mV) of the EAG response to compound; EAG(ctl1) is the EAG response to control at the beginning of the recording; EAG(ctl2) is the EAG response to control at the end of the recording; EAG(std1) is the EAG response to standard at the beginning of the recording; EAG (std2) is the EAG response to standard at the end of the recording.

First, we compared the normalized EAG-responses to selected compounds between the sexes with two-way ANOVA, followed by multiple comparisons corrected with the Bonferroni test (Prism 5, Graphpad Software). Second, the mean EAG responses relative to the standard at different concentrations of each compound were analyzed by two-way ANOVA, followed by multiple comparisons with Fisher’s LSD test (Prism 5, Graphpad Software). Third, we checked for significant differences in EAG response among tested compounds at 10^−1^ and 10^−2^ by Tukey’s multiple comparisons test (Prism 5, Graphpad Software).

In the behavioral tests, the time period s *D*. *helophoroides* stayed (BSD) in each zone (5, 10 and 15 min) were compared using a multiple t-test (Prism 5, Graphpad Software). Additionally, the frequencies of the beetles entering (BEF) each zone during 15 min of testing were compared using a chi-square test (SPSS Statistics 22, IBM). The TDM, BEF and BSD response measurements by *D*. *helophoroides* in the olfactometer were normalized and compared using one-way ANOVA, followed by Tukey’s multiple comparisons test (Prism 5, Graphpad Software).

## Results

### EAG responses of *D*. *helophoroides* to compounds from cerambycid-attacked trees

**Fig 2:**
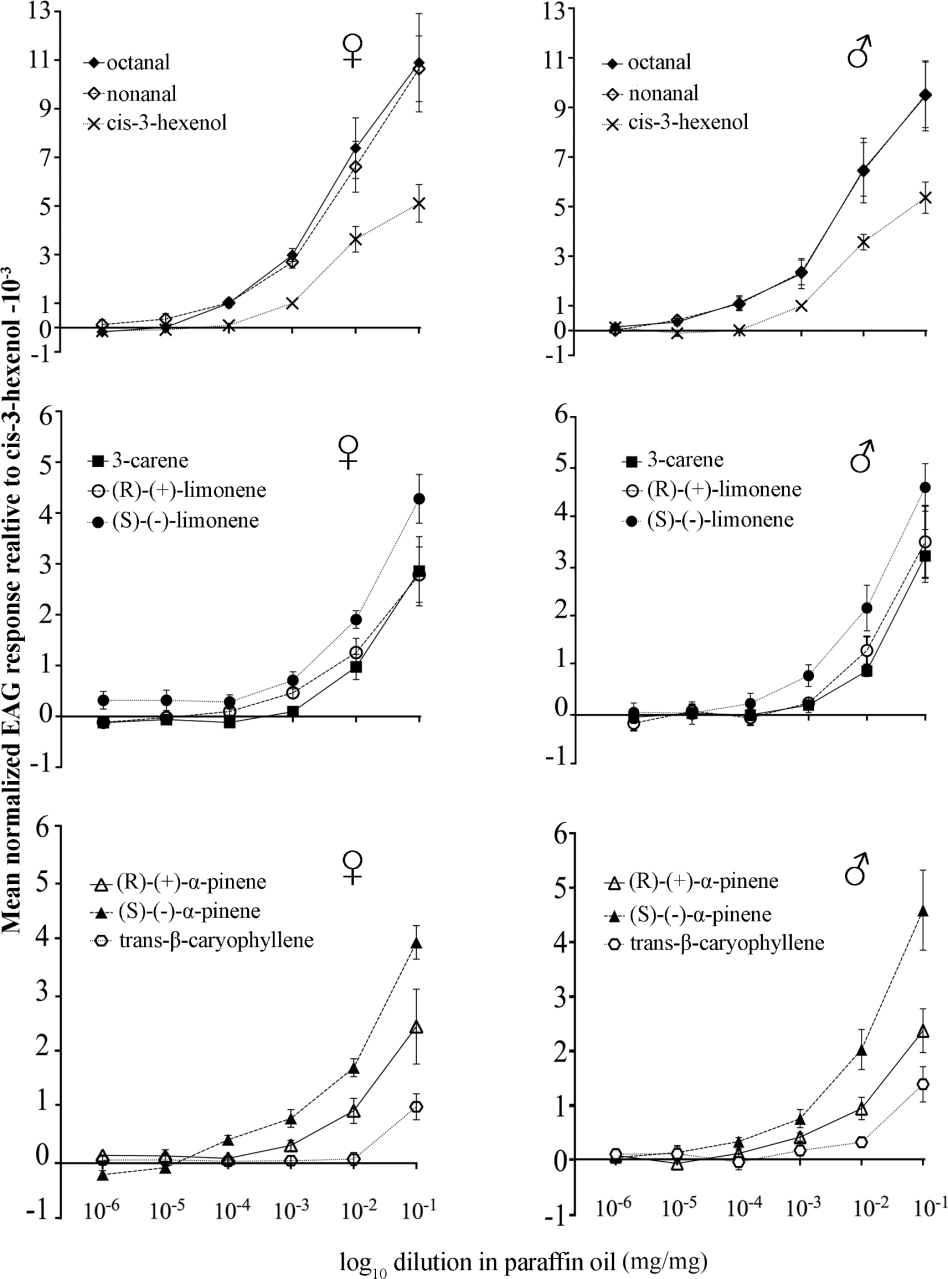
Normalized EAG responses of both female and male *D*. *helophoroides* to selected volatile compounds. The error bars represent the mean standard error (n = 10).

The normalized EAG responses of female and male *D*. *helophoroides* to nonanal, octanal, cis-3-hexenol, 3-carene, (R)-(+)-limonene, (S)-(-)-limonene, (R)-(+)—α-pinene, (S)-(-)-α-pinene and trans-β-caryophyllene at six doses (10^−1^–10^−6^ diluted in paraffin oil mg/mg) are shown in Fig 2. Of these, the compounds octanal, nonanal and (S)-(-)-limonene elicited clear dose-dependent EAG-responses down to 10^−6^ dose in both sexes. On the other hand, the common plant sesquiterpene trans-β-caryophyllene did not elicit the antennal response at lower doses (10^−3^, 10^−4^ and 10^−5^) in either sex of the beetles (Fig 2). The compound (S)-(-)-α-pinene elicited dose-dependent EAG responses down to 10^−6^ dose in male beetles only. However, female and male beetles did not show any significant sex specific differences between the amplitudes of their EAG responses (P>0.05, Tukey’s multiple comparisons test) (Fig 2; Table 2).

**Table 2:** Normalized mean EAG responses of *D*. *helophoroides* to different compounds of the same concentration.

Normalized mean EAG response to different compounds (diluted in paraffin oil mg/ mg)												
Volatile compounds	10^−1^		10^−2^		10^−3^		10^−4^		10^−5^		10^−6^	
	Female	Male	Female	Male	Female	Male	Female	Male	Female	Male	Female	Male
octanal	**10.89± 2.02 a**	**9.47± 1.41 a**	**7.38± 1.25 a**	**6.50± 1.08 a**	**2.98± 0.27 a**	**2.30± 0.60 a**	1.01± 0.18 a	1.13±0.28 a	0.01± 0.13 a	0.35± 0.15 a	0.18±0.23 a	0.15± 0.20 a
nonanal	**10.65± 1.35 a**	**9.51± 1.32 a**	**6.62± 1.04 a**	**6.46± 1.30 a**	**2.70± 0.25 a**	**2.35± 0.50 a**	1.02± 0.14 a	1.08±0.28 a	0.35± 0.22 a	0.43± 0.16 a	0.12± 0.19 a	0.01± 0.12 a
cis-3-hexenol	5.11± 0.77 b	5.36± 0.63 b	3.63± 0.53 b	3.57± 0.32 b	1.00± 0.00 b	1.00± 0.00 b	0.09± 0.09 a	0.01±0.06 a	0.09± 0.05 a	0.11± 0.12 a	0.14± 0.05 a	0.12± 0.11 a
3-carene	2.86± 0.68 c	3.20± 0.53 c	0.98± 0.25 cd	**0.89± 0.11 d**	0.09± 0.08 b	0.20± 0.15 b	0.12± 0.11 a	0.00±0.12 a	0.06± 0.07 a	0.03± 0.11 a	0.12± 0.05 a	0.04± 0.13 a
(R)-(+)-limonene	2.79± 0.54 c	3.49± 0.73 d	1.26± 0.28 cd	1.30± 0.28 cd	0.46± 0.10 b	0.24± 0.11 b	0.10± 0.07 a	0.06±0.15 a	0.02± 0.04 a	0.08± 0.14 a	0.12± 0.07 a	0.17± 0.15 a
(S)-(-)-limonene	4.28± 0.48 b	4.58± 0.48 d	1.91± 0.17 c	2.15± 0.46 c	0.71± 0.17 b	0.79± 0.22 b	0.29± 0.14 a	0.23±0.20 a	0.32± 0.20 a	0.04± 0.23 a	0.32± 0.17 a	0.05± 0.19 a
(R)-(+)-α-pinene	2.43± 0.67 c	2.37± 0.40 c	0.93± 0.22 cd	0.93± 0.21 cd	0.32± 0.09 b	0.41± 0.08 b	0.08± 0.03 a	0.11±0.08 a	0.13± 0.11 a	0.08± 0.08 a	0.14± 0.07 a	0.07± 0.13 a
(S)-(-)-α-pinene	3.93± 0.30 bc	4.58± 0.74 d	1.70± 0.16 c	2.02± 0.37 cd	0.80± 0.16 b	0.75± 0.17 b	0.42± 0.08 a	0.32±0.08 a	0.08± 0.09 a	0.12± 0.13 a	0.2± 0.07 a	0.03± 0.09 a
trans-β-caryophyllene	**1.00± 0.23 d**	**1.38± 0.32 e**	**0.07± 0.12 d**	**0.31± 0.10 d**	0.04± 0.09 b	0.16± 0.15 b	0.03± 0.08 a	0.05±0.14 a	0.06± 0.11a	0.10± 0.13 a	0.06± 0.06 a	0.09± 0.10 a

Different letters indicate significant differences among different compounds (P<0.05, Tukey’s multiple comparisons test at the same concentration level). Values are means (± standard error). (n = 10).

When comparing the normalized EAG responses of both sexes of *D*. *helophoroides* to all nine volatile compounds at the two highest doses (10^−1^ and 10^−2^) (Table 2), the highest EAG response to the aldehydes octanal and nonanal was recorded for female beetles at a dose of 10^−1^ (P<0.05, Tukey’s multiple comparisons test). In contrast, at a dose of 10^−2^, both sexes showed significantly higher EAG response amplitudes to octanal and nonanal than to the other tested compounds (Table 2) (P<0.05, Tukey’s multiple comparisons test). The EAG response amplitudes of both sexes to trans-β-caryophyllene at 10^−1^ and 10^−2^ were significantly lower than to all other compounds (Table 2) (P<0.05, Tukey’s multiple comparisons test).

### Behavioral responses of *D*. *helophoroides* to selected volatile compounds

#### Total distance movement (TDM)

We calculated the total distance moved of *D*. *helophoroides* as a combined behavioral response to different concentrations (10^−1^, 10^−3^, 10^−5^ diluted in silicone oil mg/mg) of all compounds and silicone oil control within 15 min (Table 3). Comparison of TDM for both sexes to all compounds at different doses relative to the control stimulus showed that the female beetles exposed to trans-β-caryophyllene at 10^−1^ exhibited a significantly increased TDM. Moreover, the TDM of female beetles was significantly reduced when exposed to a 10^−5^ dose of cis-3-hexenol. However, both sexes exposed to 3-carene and (R)-(+)-limonene showed significantly increased TDM at high (10^−1^) doses compared to low (10^−5^). Male beetles showed this effect to trans-β-caryophyllene even at a dose of 10^−1^ relative to 10^−3^ dose (Table 3).

**Table 3:** Total distance movement (TDM) in 15 min by *D*. *helophoroides* exposed to different concentrations (10^−1^, 10^−3^, 10^−5^) diluted in silicone oil mg/mg) of volatile compounds.

Volatile compounds	Total distance moved in 15 min (cm)					
	Female			Male		
	10^−1^	10^−3^	10^−5^	10^−1^	10^−3^	10^−5^
Octanal	20.23±2.52 cd	20.89±2.17 cd	17.74±2.21 cd	18.33±2.37 cd	19.56±1.80 cd	24.89±3.68 bc
nonanal	22.11±2.01 bc	27.24±1.62 b	22.13±2.42 bc	24.53±2.04 bc	26.85±2.03 bc	22.90±3.07 bc
cis-3-hexenol	20.24±2.12 cd	22.13±2.36 bc	**15.73±1.48 d**	16.31±1.26 cd	18.62±1.65 cd	16.62±1.18 cd
3-carene	31.70±2.77 ab	21.31±1.94 bc	18.60±1.22 cd	30.90 ±3.72 ab	21.46±2.18 bc	19.73±1.35 cd
(R)-(+)-limonene	28.71±3.56 ab	27.17±5.37 bc	17.84±1.40 cd	30.73±3.36 ab	23.05±2.41 bc	16.13±1.33 cd
(S)-(-)-limonene	19.86±1.43 cd	21.30±1.74 c	-	20.91±1.27 cd	22.10±1.95 bc	-
(R)-(+)-α-pinene	25.45±1.82 bc	30.65±2.29 ab	-	24.43±2.55 bc	24.29±2.32 bc	-
(S)-(-)-α-pinene	19.76±1.45 cd	22.76±1.93 bc	-	19.23±1.40 cd	22.17±1.66 bc	-
trans-β-caryophyllene	**34.07±5.31 a**	23.93±2.70 bc	-	30.39±4.84 ab	23.71±2.32 bc	-
silicone oil control	23.13±17.34 bc			22.24±18.59 bc		

Different letters indicate significant difference among different compounds (P<0.05, one-way ANOVA followed by Tukey’s multiple comparisons test). Values are means (± standard error). n = 50

#### Beetle entering frequency (BEF) into the selected zones

**Fig 3:**
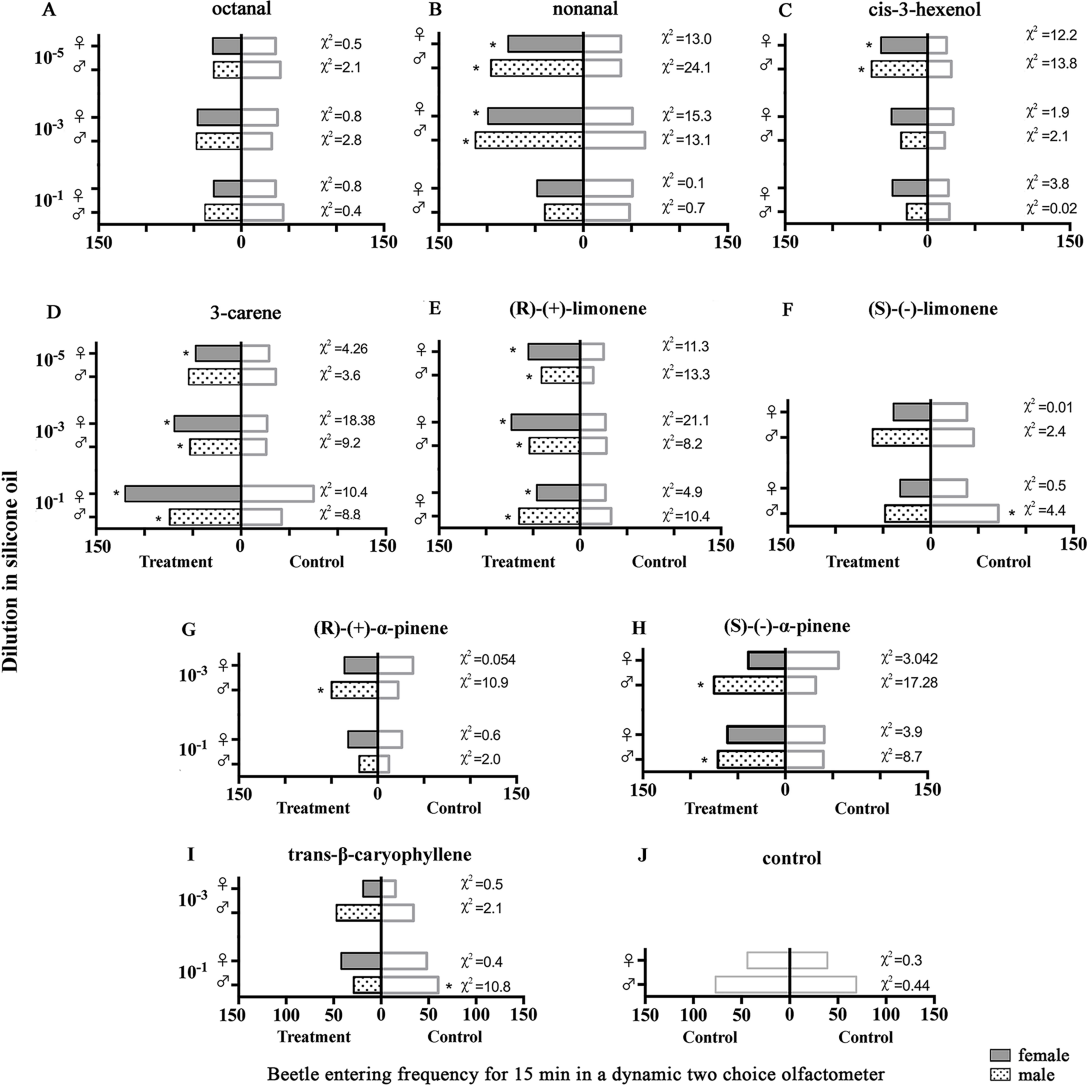
Beetle entering frequency (BEF) of *D*. *helophoroides* to treatment- and silicone oil control- zones within 15 min of exposure to different concentrations of volatile compounds diluted in silicone oil (mg/mg). Left bars indicate entering frequency to treatment zones, right bars indicate entering frequency to control zones. Asterisk indicate significant differences among different treatments (P < 0.05, chi-square test, n = 50). Control- silicone oil.

To see the effect of volatile compounds on *D*. *helophoroides* behavior, we calculated the sum of entering frequencies for each treatment- and silicone oil control- zones over a 15 min stimulus period (Fig 3). We found that both sexes exposed to 3-carene and (R)-(+)-limonene showed significantly higher (P < 0.05, chi-square test) BEF than silicone oil alone at nearly all tested doses (10^−1^, 10^−3^, 10^−5^ diluted in silicone oil mg/mg) (Fig 3). Similarly, the compound nonanal at 10^−3^, 10^−5^ and cis-3-hexenol at a dose of 10^-5^elicited significantly increased BEF for both sexes into the treatment zone compared to the control zone (P < 0.05, chi-square test) (Fig 3). However, only male beetles showed significantly increased BEF into the zones treated with (R)-(+)-α-pinene at 10^−3^ and (S)-(-)-α-pinene at 10^−1^ and 10^−3^ doses (Fig 3) compared to control zones. In contrast, the BEF of male beetles into control zones was significantly higher compared to zones treated with (S)-(-)-limonene and trans-β-caryophyllene at a dose of 10^−1^ (Fig 3). Neither sex of *D*. *helophoroides* showed any significant differences in BEF when silicone oil was tested *versus* silicone oil (P > 0.05, chi-square test) (Fig 3). The overall comparisons of BEFs for both sexes over a 15 min interval to all tested compounds at different doses (10^−1^, 10^−3^, 10^−5^ diluted in silicone oil mg/mg) are presented in Table 4.

**Table 4:** Beetle entering frequency (BEF) in 15 min by *D*. *helophoroides* exposed to different concentrations (10^−1^, 10^−3^, 10^−5^) diluted in silicone oil mg/mg) of volatile compounds.

**Volatile compounds**	Beetle entering frequency in 15 min (number of times)					
	Female			Male		
	10^−1^	10^−3^	10^−5^	10^−1^	10^−3^	10^−5^
Octanal	0.58±0.14 b	0.92±0.19 b	0.60±0.11 b	0.76±0.20 b	0.94±0.36 b	0.58±0.12 b
nonanal	0.96±0.19 b	**1.98±0.26 a**	**1.56±0.24 a**	0.80±0.12 b	**2.24±0.24 a**	**1.92±0.33 a**
cis-3-hexenol	0.74±0.20 b	0.76±0.19 b	0.98±0.17 b	0.44±0.11 b	0.56±0.14 b	1.18±0.21 b
3-carene	**2.40±0.34 a**	**1.38±0.39 a**	0.94±0.20 b	1.48±0.22 ab	1.08±0.21 b	1.08±0.13 b
(R)-(+)-limonene	0.92±0.15 b	**1.46±0.27 a**	1.10±0.25 b	**1.30±0.23 a**	1.08±0.20 b	0.82±0.14 b
(S)-(-)-limonene	0.64±0.12 b	0.78±0.11 b	-	0.96±0.28 b	**1.22±0.29 a**	-
(R)-(+)-α-pinene	0.64±0.14 b	0.72±0.13 b	-	0.40±0.11 b	1.00±0.29 b	-
(S)-(-)-α-pinene	**1.22±0.20 a**	0.78±0.11 b	-	**1.42±0.31 a**	1.50±0.29 ab	-
trans-β-caryophyllene	0.84±0.17 b	0.38±0.09 b	-	0.58±0.16 b	0.94±0.22 b	-
silicone oil control	0.78±0.15 b			**1.38±0.20 a**		

Different letters indicate significant difference among different compounds (P<0.05, one-way ANOVA followed by Tukey’s multiple comparisons test). Values are means (± standard error). n = 50

#### Beetle staying duration (BSD) in the selected zones

**Fig 4:**
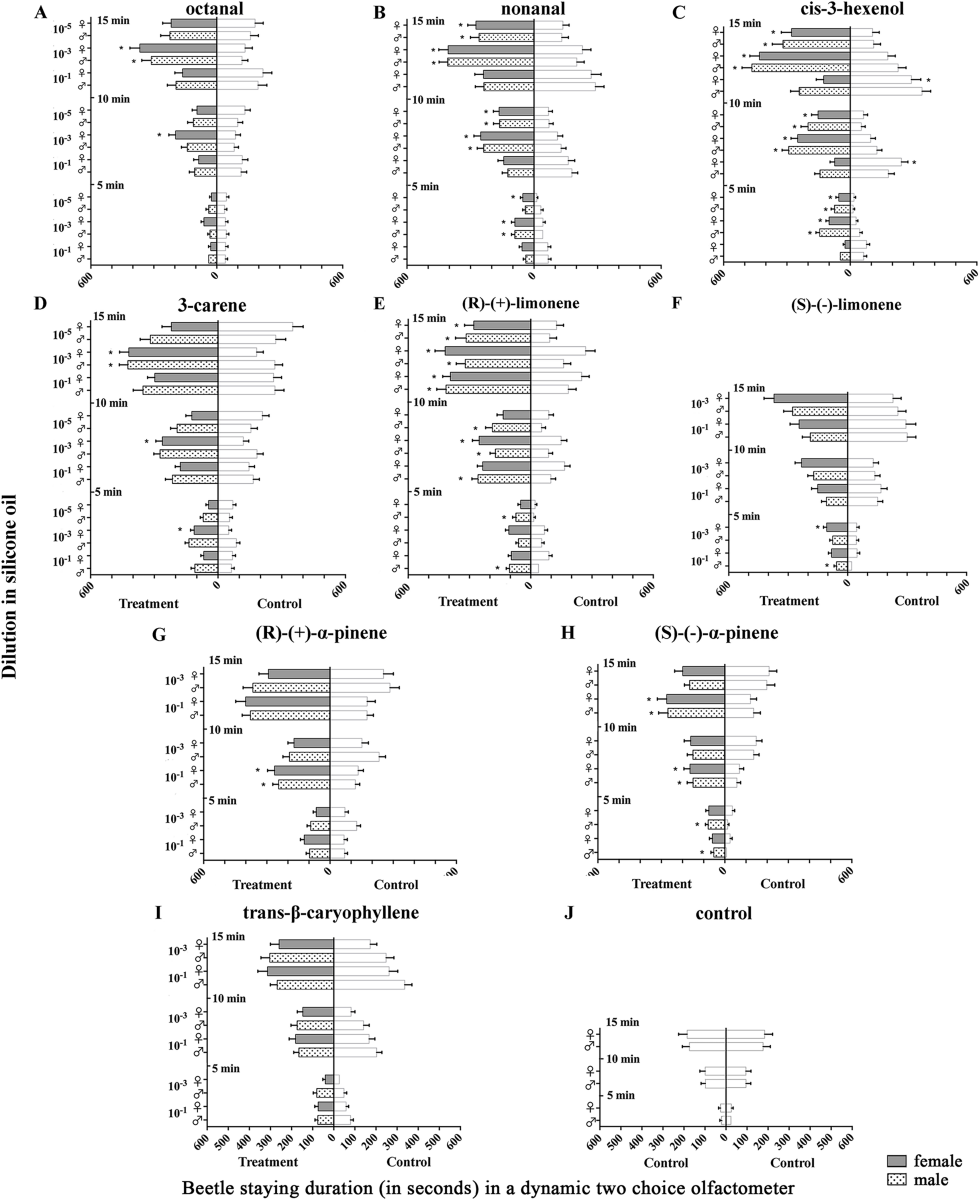
Beetle staying duration (BSD) (in seconds) of both sexes of *D*. *helophoroides* in treatment and silicone oil control zones within 5, 10 and 15 min of exposure to different concentrations (diluted in silicone oil mg/mg) of volatile compounds. Left bars indicate staying duration in treatment zones, right bars indicate staying duration in control zones. Asterisk indicate significant differences among different treatments (P < 0.05, multiple t-test). The error bars represent the mean standard error (n = 50). Control- silicone oil.

In order to measure the attractive behavior of *D*. *helophoroides* to all tested compounds at different doses (10^−1^, 10^−3^, 10^−5^ diluted in silicone oil mg/mg), we compared the mean beetle staying durations at 5, 10 and 15 min stimulus periods in treatment- and silicone oil control- zones (Fig 4). In both sexes at 5 min, the volatile compounds nonanal, cis-3-hexenol, 3-carene, (R)-(+)-limonene and (S)-(-)-α-pinene elicited significantly increased BSD in treatment zones (P< 0.05, t-test) compared to control zones mostly at lower doses (10^−3^, 10^−5^) (Fig 4). Similarly, beetles exposed to different concentrations of compounds for 10 and 15 min periods revealed that the BSD in zones treated with nonanal, cis-3-hexenol, and (R)-(+)-limonene were significantly increased (P< 0.05, t-test) compared to control zones at a lower dose in both sexes of the beetles (Fig 4). Additionally, higher concentrations (10^−1^) of (R)-(+)-α-pinene and (S)-(-)-α-pinene elicited significantly increased BSD at 10 min and at 5, 10 and 15 min respectively in treated zones compared to control zones of both sexes (Fig 4). In contrast, female beetles exposed to cis-3-hexenol at a higher dose (10^−1^) for 10 and 15 min showed significantly higher BSD in the control zones (Fig 4). Neither sex tested in silicone oil *versus* silicone oil showed any significant difference in BSD at any tested period (Fig 4) (P> 0.05, t-test). The overall comparisons of BSD for both sexes within 5, 10 and 15 min to all compounds at different doses (10^−1^, 10^−3^, 10^−5^ diluted in silicone oil mg/mg) are shown in Tables 5–7.

**Table 5:** Beetle staying duration (BSD) in 5 min by *D*. *helophoroides* exposed to different concentrations (10^−1^, 10^−3^, 10^−5^) diluted in silicone oil mg/mg) of volatile compounds.

Volatile compounds	Beetle staying duration in 5 min (in seconds)					
	Female			Male		
	10^−1^	10^−3^	10^−5^	10^−1^	10^−3^	10^−5^
octanal	28.98±10.02 b	61.19±13.31 b	25.57±8.56 b	38.84±11.07 b	32.76±10.08 b	38.84±12.39 b
nonanal	56.80±13.30 b	90.48±15.76 ab	52.84±11.49 b	39.23±11.34 b	90.94±15.55 ab	41.73±11.5 b
cis-3-hexenol	24.64±8.54 b	100.06±15.16 ab	54.18±13.60 b	47.33±10.57 b	**144.81±17.59 a**	74.97±15.5 ab
3-carene	67.21±13.52 ab	112.35±16.91 ab	44.30±12.68 b	109.29±17.56 ab	137.03±17.66 ab	69.55±13.96 ab
(R)-(+)-limonene	94.95±14.67 ab	107.09±16.19 ab	48.98±13.35 b	104.05±15.61 ab	60.13±12.36 b	72.86±15.59 ab
(S)-(-)-limonene	81.31±15.85 ab	105.55±16.34 ab	-	56.30±12.25 b	75.34±14.78 ab	-
(R)-(+)-α-pinene	122.33±17.92 ab	65.73±3.82 b	-	97.26±5.84 ab	91.9±17.07 ab	-
(S)-(-)-α-pinene	59.17±13.48 b	75.64±14.40 ab	-	52.36±13.54 b	78.84±13.02 ab	-
trans-β-caryophyllene	74.11±15.54 ab	40.3±11.57 b	-	75.69±13.43 ab	80.85±16.02 ab	-
silicone oil control	25.39±8.63 b			23.49±8.80 b		

Different letters indicate significant difference among different compounds (P<0.05, one-way ANOVA followed by Tukey’s multiple comparisons test). Values are means (± standard error). n = 50

**Table 6:** Beetle staying duration (BSD) in 10 min by *D*. *helophoroides* exposed to different concentrations (10^−1^, 10^−3^, 10^−5^) diluted in silicone oil mg/mg) of volatile compounds.

Volatile compounds	Beetle staying duration in 10 min (in seconds)					
	Female			Male		
	10^−1^	10^−3^	10^−5^	10^−1^	10^−3^	10^−5^
octanal	86.51±23.54 b	196.52±31.98 ab	94.42±22.68 b	105.75±24.93 b	140.48±27.77 ab	111.73±27.16 b
nonanal	142.96±27.13 ab	251.69±31.96 ab	164.99±26.65 ab	124.30±25.57 b	238.05±30.15 ab	163.30±27.89 ab
cis-3-hexenol	73.92±21.51 b	249.36±31.23 ab	152.54±30.44 ab	143.62±24.40 ab	**291.57±31.10 a**	200.02±33.17 ab
3-carene	177.41±24.13 ab	262.29±30.23 ab	123.33±28.30 b	214.59±33.05 ab	272.94±29.05 ab	192.67±30.63 ab
(R)-(+)-limonene	235.16±26.08 ab	252.48±32.94 ab	135.09±29.43 ab	257.91±30.23 ab	173.17±25.91 ab	187.39±33.38 ab
(S)-(-)-limonene	151.03±31.11 ab	232.82±32.40 ab	-	107.46±24.38 b	171.59±29.52 ab	-
(R)-(+)-α-pinene	263.40±33.31 ab	171.19±28.80 ab	-	244.06±27.48 ab	192.84±30.93 ab	-
(S)-(-)-α-pinene	164.66±28.52 ab	161.36±30.54 ab	-	150.65±27.27 ab	150.95±26.42 ab	-
trans-β-caryophyllene	181.20±30.36 ab	146.94±25.92 ab	-	165.35±24.10 ab	173.88±28.77 ab	-
*silicone oil control*	94.79±24.31 b			95.13±22.57 b		

Different letters indicate significant difference among different compounds (P<0.05, one-way ANOVA followed by Tukey’s multiple comparisons test). Values are means (± standard error). n = 50

**Table 7:** Beetle staying duration (BSD) in 15 min by *D*. *helophoroides* exposed to different concentrations (10^−1^, 10^−3^, 10^−5^) diluted in silicone oil mg/mg) of volatile compounds.

Volatile compounds	Beetle staying duration in 15 min (in seconds)					
	Female			Male		
	10^−1^	10^−3^	10^−5^	10^−1^	10^−3^	10^−5^
octanal	162.63±36.97 c	366.74±47.08 ab	217.5±39.92 bc	194.62±40.85 c	311.90±45.46 bc	223.51±43.52 bc
nonanal	238.76±40.73 bc	405.44±44.34 ab	274.33±38.67 bc	237.24±39.78 bc	407.80±40.60 ab	259.56±40.67 bc
cis-3-hexenol	125.84±35.41 c	430.15±45.51 ab	278.20±48.5 bc	242.12±40.1 bc	**466.14±46.39 a**	318.91±50.40 b
3-carene	297.62±33.9 bc	419.82±43.26 ab	219.82±43.77 bc	353.40±45.97 ab	424.94±39.79 ab	318.63±47.59 b
(R)-(+)-limonene	393.58±37.22 ab	419.28±49.11 ab	278.82±44.86 bc	414.84±37.22 ab	320.65±44.19 b	316.02±52.27 b
(S)-(-)-limonene	244.85±45.83 bc	371.58±50.45 ab	-	188.83±39.44 c	278.06±44.67 bc	-
(R)-(+)-α-pinene	400.34±47.19 ab	292.44±44.74 bc	-	378.27±38.95 ab	366.53±45.9 ab	-
(S)-(-)-α-pinene	274.80±44.06 bc	199.08±37.85 c	-	269.58±42.99 bc	166.55±24.55 c	-
trans-β-caryophyllene	313.99±45.93 b	258.83±41.17 bc	-	268.29±32.4 bc	303.41±41.19 bc	-
*silicone oil control*	183.90±37.78 c			176.66±34.21 c		

Different letters indicate significant difference among different compounds (P<0.05, one-way ANOVA followed by Tukey’s multiple comparisons test). Values are means (± standard error). n = 50

## Discussion

Previous results from our laboratory showed that the antenna of *D*. *helophoroides* contains different types of sensilla with a porous cuticle, suggesting that these sensilla might be involved in olfaction [11]. In the present study, the EAG responses of *D*. *helophoroides* were recorded to explore the antennal detection of their prey-associated volatile compounds [5, 14–16, 9, 10, 22]. To the best of our knowledge, this study is the first to investigate both electrophysiological and behavioral responses of *D*. *helophoroides* to their prey-associated volatile compounds at different concentrations. The aldehydes octanal and nonanal eliciting the highest EAG response amplitudes in both sexes of the beetles suggesting that more olfactory chemoreceptors are involved in the detection of these aldehydes [23]. However, in our behavioral tests, both sexes showed statistically significant responses only to nonanal. This shows that highly EAG-active compounds do not always necessarily elicit behavioral responses. Nevertheless, mixtures of these aldehydes might elicit significant behavioral responses by *D*. *helophoroides* in an olfactometer as demonstrated by their prey *A*. *glabripennis* [24].

Both, female and male antennae showed high EAG responses to cis-3-hexenol. However, different concentrations of this compound have different effects on the behavioral responses of the two sexes. For example, female beetles were significantly repelled when exposed to cis-3-hexenol at a dose of 10^−1^, whereas at a dose of 10^−3^, the same compound was attractive. Even an attractive effect was elicited by cis-3-hexenol at a dose of 10^−5^ for both female and male beetles, suggesting a role in finding mating partners and oviposition substrates (Table 8). Moreover, this green leaf volatile is released from damaged deciduous trees, eliciting strong EAG and attractive behavioral responses to *B*. *horsfieldi* and *A*. *glabripennis*, prey species of *D*. *helophoroides* **[25–27]**. Thus, cis-3-hexenol might serve as a kairomone for deciduous trees by providing a clue to the cerambycid herbivores and serve as a synomone by luring predators of these herbivorous beetles.

**Table 8:** Different behavioral response patterns of *D*. *helophoroides* to selected volatile compounds at different concentrations (10^−1^, 10^−3^, 10^−5^ diluted in silicone oil mg/mg) as determined by the TDM, BEF and BSD assay parameters.

Chemicals	Concentration (mg/mg)	TDM	BEF	BSD	Behavior response	
					Female	Male
Octanal	10^−1^	-	-	-	-	-
	10^−3^	-	-	F+M+	Att.	Att.
	10^−5^	-	-	-	-	-
nonanal	10^−1^	-	-	-	-	-
	10^−3^	-	F+M+	F+M+	H. Att.	H. Att.
	10^−5^	-	F+M+	F+M+	H. Att.	H. Att.
cis-3-hexenol	10^−1^	-	-	F-	Rep.	-
	10^−3^	-	-	F+M+	Att.	Att.
	10^−5^	F-	F+M+	F+M+	H. Att.	H. Att.
3-carene	10^−1^	F+M+	F+ M+	-	Att.	Att.
	10^−3^	-	F+M+	F+M+	H. Att.	H. Att.
	10^−5^	-	F+	-	Att.	-
(R)-(+)-limonene	10^−1^	F+M+	F+M+	F+M+	H. Att.	H. Att.
	10^−3^	-	F+M+	F+M+	H. Att.	H. Att.
	10^−5^	-	F+M+	F+M+	H. Att.	H. Att.
(S)-(-)-limonene	10^−1^	-	M-	-	-	Rep.
	10^−3^	-	-	-	-	-
(R)-(+)-α-pinene	10^−1^	-	-	F+M+	Att.	Att.
	10^−3^	F+	M+	-	RM	Att.
(S)-(-)-α-pinene	10^−1^	-	M+	F+M+	Att.	Att.
	10^−3^	-	M+	-	-	Att.
trans-β-caryophyllene	10^−1^	F+M+	M-	-	RM	Rep.
	10^−3^	-	-	-	-	-

**‘TDM’** Total Distance Movement; **‘BEF’** Beetle Entering Frequency; **‘BSD’** Beetle Staying Duration. **‘-’** no significant response; **‘F+’** significant positive response of female; **‘M+’** significant positive response of male; **‘F-’** significant negative response of female; **‘M-’** significant negative response of male.

F+/M+ in TDM- robust movement towards tested zones in the same track (does not alone reveal the kinesis activity of the beetles).

F-/M- in TDM- little movement towards any tested zones in the same track (does not alone reveal the kinesis activity of the beetles).

F+/M+ in BEF- Attraction

F-/M- in BEF- Repulsion

F+/M+ in BSD- Attraction

F-/M- in BSD- Repulsion

**Att**.- Attraction, **H. Att**.- High Attraction (**Att.** in both BEF and BSD), **Rep**.- Repulsion, **RM.**- Robust Movement, **LM.**- Little Movement.

The common plant sesquiterpene trans-β-caryophyllene elicited a weak EAG response and a strong repellent behavior in both female and male beetles. This is underlined by significantly increased TDM response of *D*. *helophoroides* when exposed to trans-β-caryophyllene suggesting that this compound may contribute to non-host avoidance.

The monoterpenes 3-carene, (R)-(+)-limonene, (S)-(-)-limonene, (R)-(+)-α-pinene and (S)-(-)-α-pinene elicited intermediate EAG responses. No significant sex specific antennal response differences to any of these compounds was observed. Moreover, behavioral responses of both sexes towards these monoterpenes are quite similar. Additionally, there was no sexual dimorphism in EAG and behavioral responses to enantiomers of α-pinene and limonene. This is not unexpected as both sexes live on resources characterized by prey associated volatile cues. Similar results have been demonstrated in the cerambycid *B*. *horsfieldi* **[25]**.

In both sexes, we found a strong discrimination between the enantiomers of limonene, both in EAG and behavioral responses of *D*. *helophoroides*. (S)-(-)-limonene elicited significantly higher EAG responses in both sexes. However, in behavioral tests only (R)-(+)-limonene elicited an attraction response in both sexes. These results are in agreement with a previous report **[10]**, which demonstrated that *D*. *helophoroides* adults were significantly attracted by (R)-(+)-limonene derived from the frass odor of the prey species *M*. *raddei*. In addition to these findings, we confirmed significant attractive responses of *D*. *helophoroides* to (R)-(+)-limonene over a broad range of concentrations. Similar enantiomeric discrimination has been observed in diverse forest tree pests like *Xylotrechus rusticus*, *Dendroctonus sp*., *Hylobius abietis* **[28–31]** as well as prey species of *D*. *helophoroides* including *A*. *glabripennis* and *M*. *alternatus* **[32, 14, 33]**. This highlights the importance of enantiomeric discrimination for finding suitable plant and/or prey. Consequently, highly specific enantiomer discrimination of prey associated compounds might sharpen the searching ability of predators like *D*. *helophoroides* to reach their prey.

The prey-associated plant volatiles played a crucial role in the prey location process of *D*. *helophoroides*, as demonstrated in another coleopteran predator *Thanasimus dubius* for the forest pest *Dendroctonus frontalis* **[29]**. *D*. *helophoroides* is an oligophagous predator that preys on at least six forest pest species of cerambycids **[2, 10]**. Thus, adult *D*. *helophoroides* need to adapt to a broad range of prey-associated chemical cues that are not necessarily specific to one prey insect. EAG and behaviorally active compounds in this beetle, such as nonanal, cis-3-hexenol, 3-carene, (R)-(+)-limonene, (R)-(+)-α-pinene and (S)-(-)-α-pinene, are common host volatiles or feeding-induced volatiles of several cerambycid beetle species **[2, 10, 34]**. For example, nonanal, cis-3-hexenol, 3-carene, (R)-(+)-α-pinene and (S)-(-)-α-pinene were previously reported to generate robust EAG responses and elicit behavioral responses to *A*. *glabripennis* and *B*. *horsfieldi* **[24–27, 35, 32]**. Similarly, (R)-(+)-α-pinene and 3-carene were also reported generate robust EAG responses in *M*. *alternatus* **[14]**. This suggests that many cerambycid beetles use host plant volatile cues for finding their host and assessing the suitability for feeding or oviposition. Similarly, an olfactory response of *D*. *helophoroides* to prey associated volatile compounds either from their prey host plant or prey frass **[9, 10, 26, 32, 35]** is viewed as an adaptive strategy to locate the prey. This also suggests that *D*. *helophoroides* is mainly relying on different prey associated volatile cues either in individual or in particular ratios to find prey, as well as for mating and oviposition site selection.

In conclusion, the EAG results of this study represent an initial attempt to demonstrate the electrophysiological sensitivity of both sexes of *D*. *helophoroides* to prey-associated plant volatiles. In addition to previous behavioral studies **[9, 10]**, we found that the beetles exhibited concentration dependent behavioral responses with different behavioral response affected/triggered. Most of the selected compounds elicited notable EAG and behavioral responses. The beetle *D*. *helophoroides* has 52 odorant binding proteins (OBPs), 19 chemosensory proteins (CSPs),10 olfactory receptors (ORs), 8 ionotropic receptors (IRs), 2 gustatory receptors (GRs), and 5 sensory neuron membrane proteins (SNMPs) in their antennae **[13]**, suggesting a differentiated role in olfaction. Our results can foster future research approaches combining electrophysiological methods with molecular techniques such as RNAi knockdown to understand the olfactory mechanism of this predatory beetle in detail. Our results show that octanal, nonanal, cis-3-hexenol, 3-carene, (S)-(-)-α-pinene and (R)-(+)-limonene are most acutely perceived by *D*. *helophoroides* antenna. These compounds might be useful either individually or in mixtures for developing efficient attractants to lure this predatory beetle into forest stands damaged by cerambycid herbivores. For instance, the green leaf volatile cis-3-hexenol is sensitively perceived by *D*. *helophoroides* but not by its prey *M*. *alternatus***[14]**. Therefore, cis-3-hexenol might be used as an allochthonous kairomone **[36]** to attract the *D*. *helophoroides* beetles into the *M*. *alternatus* attacked forest stands without attracting more *M*. *alternatus*. Additionally, the most effective compounds should be tested in various combinations in the field trapping experiments or in biological control sites to see if the compounds increase the attraction of *D*. *helophoroides* and by extension enhance predation rates.
